# Effects of vitamin A on intramuscular fat development in beef cattle: A meta-analysis

**DOI:** 10.3389/fvets.2023.1105754

**Published:** 2023-03-15

**Authors:** Wei Li, Fang Wang, Fang Sun, Yongli Qu, Chunhai Liu, Yongsheng Han, Hongbao Wang, Botao Jiang, Peng Zhong, Jiahui Wang, Xueying Song, Meng Huang, Deli Ding

**Affiliations:** ^1^Heilongjiang Academy of Agricultural Sciences Livestock Veterinary Branch, Qiqihar, Heilongjiang, China; ^2^Heilongjiang Provincial Key Laboratory of Resistance Gene Engineering and Protection of Biodiversity in Cold Areas, College of Life Sciences and Agroforestry, Qiqihar University, Qiqihar, Heilongjiang, China; ^3^Institute of Animal Husbandry, Heilongjiang Academy of Agricultural Sciences, Harbin, China; ^4^College of Animal Science and Technology, Heilongjiang Bayi Agricultural University, Daqing, Heilongjiang, China; ^5^Liaoning FEEDIG Feedstuff Technology Co., Ltd., Xingcheng, Liaoning, China

**Keywords:** vitamin A, intramuscular fat, beef cattle, steers, meta-analysis

## Abstract

Vitamin A, a fat-soluble vitamin, is the basic substance required to maintain healthy vision and the main physiological functions of cattle. The results from previous studies regarding the effect of vitamin A on intramuscular fat varied. This meta-analysis aimed to generate a more comprehensive understanding of the relationship between vitamin A and intramuscular fat content and to provide potential clues for future research and commercial practice. Electronic databases such as MEDLINE and Ovid were systematically searched, and studies investigating the relationship between vitamin A and intramuscular fat content were included. Standardized mean differences (SMDs) in intramuscular fat percentage and intramuscular fat score, with their respective 95% confidence intervals (CIs), were calculated. The heterogeneity and publication bias were evaluated. A total of 152 articles were identified through searches of databases. Seven articles were confirmed for inclusion in this meta-analysis. The SMD of IMF percentage derived from the analysis was−0.78 (-2.68, 1.12) (Q = 246.84, *p* < 0.01). The SMD of the IMF score was 1.25 (-2.75, 5.25) (Q = 87.20, *p* < 0.01). Our meta-analysis indicates that the addition of vitamin A could decrease intramuscular fat in cattle steers.

## Introduction

With improvement in their living standards, people expect good quality beef replete with taste-enhancing features, such as a lean meat rate, nice meat flavor, and sensory satisfaction. Marbling, also known as intramuscular fat, is a collection of white stripes and spots on the surface of a beef muscle (1). The content of beef intermuscular fat has a direct impact on the quality of beef, as it affects its juiciness, tenderness, and flavor (2, 3). When a certain amount of fat is deposited between muscle bundles and muscle fibers, the section of beef is said to have been marbled. The higher the marbling grade, the better the quality of the meat. Vitamin A, a fat-soluble vitamin, is the basic substance required to maintain healthy vision and the main physiological functions of cattle (4). Ruminants can absorb vitamin A from plant sources (such as

carotenoids or provitamin A) and feed additives (such as retinol or retinyl ester or preformed vitamin A) (5). Many physiological functions of vitamin A are realized through the retina and retinoic acid (4). Vitamin A is involved in the formation of nocturnal rhodopsin (rhodopsin), which is necessary for the functioning of normal epithelial cells. Vitamin A and carotenoids show antioxidant activity by scavenging peroxy free radicals and singlet oxygen (6, 7).

Vitamin A has been found to be negatively correlated with the marbling score of Japanese black cattle carcass for the first time (8). Similar studies were conducted in other types of cattle, such as Angus steers, Simmental steers, and Korean native steers (9–18). Some studies have shown that limiting vitamin A intake increases marbling, but other studies have shown that limiting vitamin A intake does not have a significant effect on marbling. Moreover, the sample sizes of these studies were limited. This meta-analysis aimed to generate a more comprehensive understanding of the relationship between vitamin A and intramuscular fat content and to provide potential clues for future research and commercial practice.

## Methods

The meta-analysis was conducted under the guidelines of the Preferred Reporting Items for Systematic Reviews and Meta-Analysis (PRISMA) rather than a self-designed protocol (19). This Review was not registered in the Cochrane database.

### Ethics statement

All included studies had already declared ethical approvals in the original articles; therefore, no ethical approval was necessary for this study.

### Search strategy and selection criteria

We conducted a comprehensive search of the electronic databases, MEDLINE and Ovid, from inception to 30 November 2021. Only articles in English language were considered.

The following strategy was used for the database research: MEDLINE: (((beef[Title/Abstract]) AND (Intramuscular fat[Title/Abstract])) AND (vitamin[Title/Abstract])). Ovid: (((beef[Title/Abstract]) AND (Intramuscular fat[Title/Abstract])) AND (vitamin[Title/Abstract])). Furthermore, the references in the articles were also screened for any potentially eligible studies. The inclusion criteria for the present study were as follows: (1) studies evaluating the effects of Vitamin A on intramuscular fat in cattle; (2) intramuscular fat percentage (IMF%) and/or intramuscular fat score were used for the measurement of intramuscular fat; (3) results for the vitamin A group and the control group were reported; and (4) the number of cattle in each group, in which mean and standard deviation of indicators were extracted or calculated. The exclusion criteria for the present study were as follows: (1) the data to be analyzed could not be extracted or calculated; (2) articles that were case reports, reviews, letters, news, conference abstracts, and studies regarding other types of animals; (3) the absence of a control group; and (4) duplication or overlap in the research animals. If studies were conducted by an identical research group, those studies with the largest sample size or those studies with the most comprehensive and detailed information were included. Cochrane collaboration's tool for assessing the risk of bias was used to judge the risk of bias for each included study. Two independent researchers (Name) undertook the literature search and study screening. Disagreements were resolved through discussions.

### Data extraction and quality assessments

Two investigators (WL and FW) independently performed the title and abstract screening of articles on the basis of the aforementioned inclusion criteria. Then, a full-text evaluation of the studies was conducted for the final inclusion. Moreover, the following information about each included study was extracted from or calculated based on details such as: first author's name, year of publication, type of steer, study design, total number of steers, weight of steers at the start, addition of vitamin A, and indicators in each group.

### Statistical analysis

We used R 4.0.2 software, Review Manager 5.3, and Microsoft Office Excel 2016 software for data collection and statistical analyses at the study level. A *p*-value of < 0.05 was considered to be statistically significant. We calculated pooled estimates of the standardized mean difference (SMD), the difference between an indicator of the experimental group (steers added with vitamin A) and that of the control group, and the respective 95% confidence intervals (CIs) of each indicator. The Cochran Q values and the *I*^2^ statistics were introduced to qualitatively and quantitatively explore the heterogeneity in the included studies. Insignificant, low, moderate, and high heterogeneities were identified to have *I*^2^ values of 0–25, 25–50, 50–75, and 75–100%, respectively (20). We created funnel plots to assess potential publication bias. Deek's method was used to statistically check the asymmetry of the funnel plot and detect publication bias. In addition, we conducted an influential analysis to evaluate the impacts of individual study on the overall results.

## Results

### Study selection and characteristics

**Figure 1:**
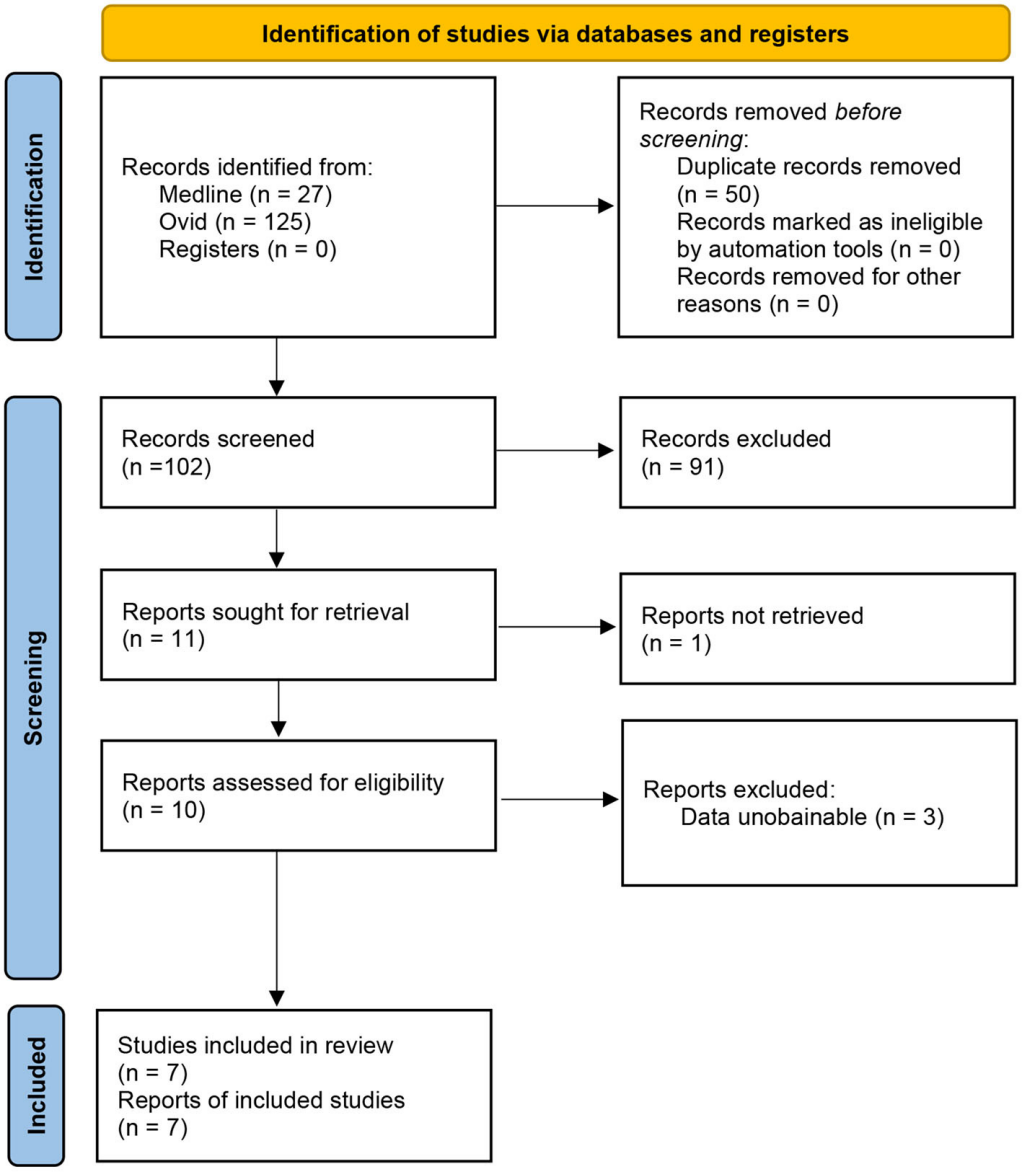
A flowchart of the literature search.

**Figure 2:**
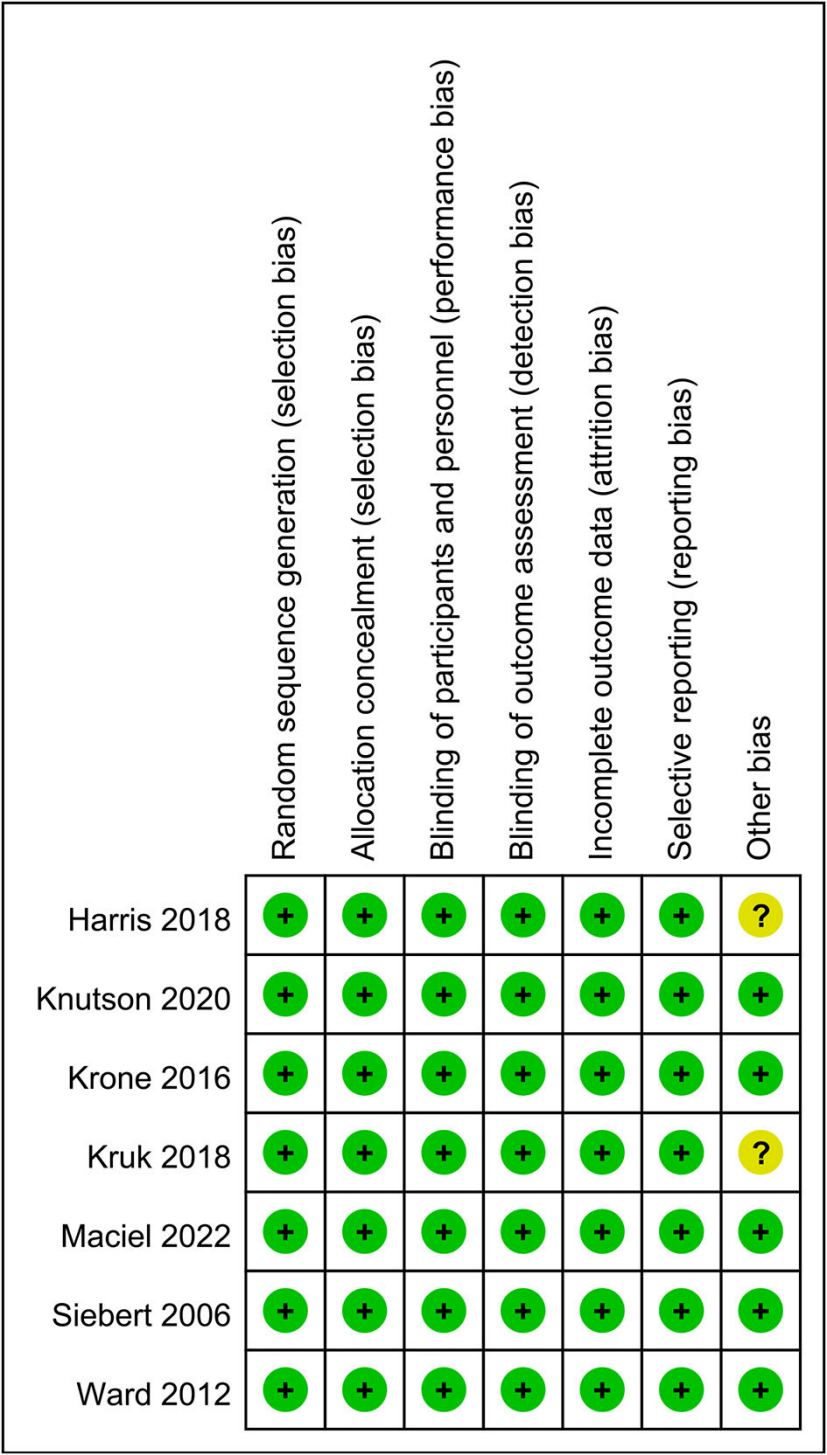
Summary of the risk of bias in the included studies.

A total of 152 articles were identified from the databases searched. Among them, 50 duplicates were removed and 91 studies were excluded through an initial screening. After a full-text assessment of the eligibility of the remaining 11 articles, four studies that might appear to have met the inclusion criteria were excluded due to limited information, eventually leaving seven articles that were identified for inclusion in this meta-analysis. No additional studies were found through bibliography screening of the included articles. Figure 1 shows a detailed flow of the database search and literature selection processes. The risk of bias for each included study was assessed as low (Figure 2; Supplementary Figure 1).

### Effect of vitamin A on IMF

**Figure 3:**
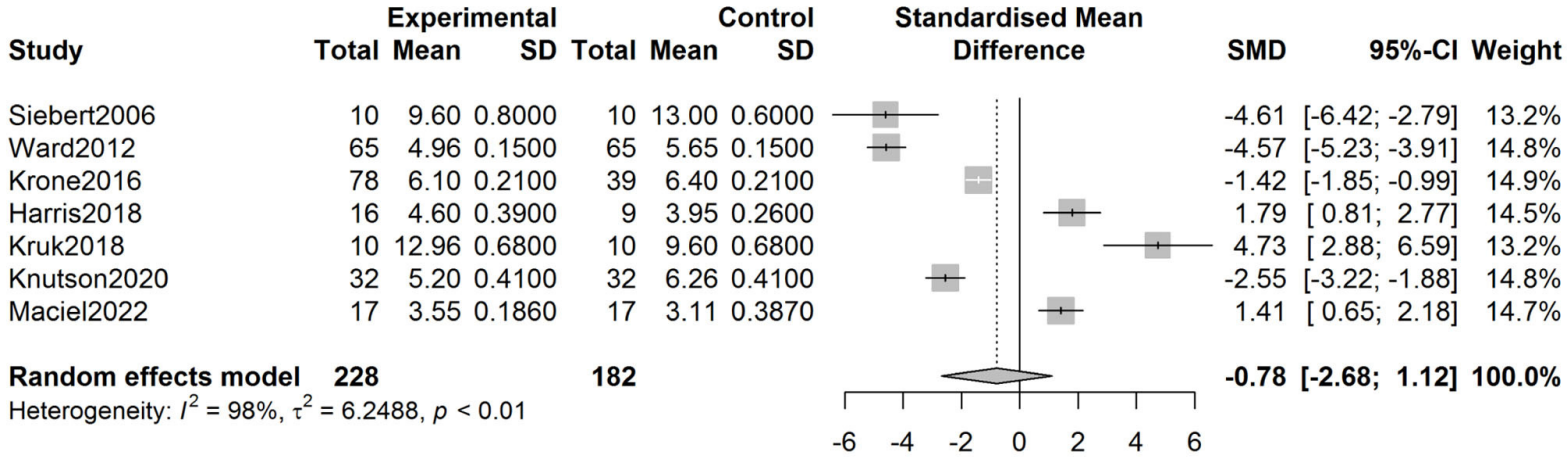
Forest plots of IMF percentage in the vitamin A and control groups.

**Figure 4:**
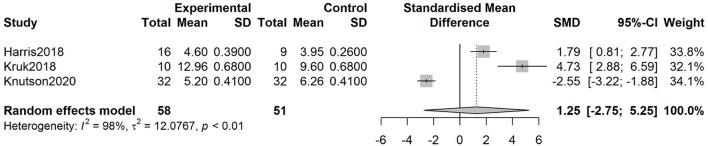
Forest plots of IMF score in the vitamin A and control groups.

The SMD of IMF percentage derived from the analysis was−0.78 (-2.68, 1.12) (*p* = 0.419) (see Figure 3). The SMD of IMF score was 1.25 (-2.75, 5.25) (*p* = 0.539) (Figure 4).

### Heterogeneity and publication bias

In the pooled analysis of IMF percentage, the test of heterogeneity product showed a Q value of 246.84 (*p* < 0.01). Heterogeneity was observed in the analysis of IMF percentage (*I*^2^ = 97.6%, *p* < 0.01). With regard to the analysis of IMF score, the Q value and *I*^2^ were 87.20 and 97.7%, respectively. Deek's tests for publication bias yielded *p*-values of 0.535 and 0.281 for the analysis of IMF percentage and IMF score, respectively.

### Sensitivity analysis

The sensitivity analysis was performed to evaluate the impacts of individual study on the overall results. No outlier was identified in all sensitivity analyses.

## Discussion

Owing to the advantages offered by high protein and low fat, beef consumption has been growing rapidly in China. However, because of limitations in variety and the production level, China's high-quality beef, especially high-grade beef, has a low output, a high cost, and a long-term dependence on beef import. Therefore, the marbling grade of beef is the main factor that determines the beef price and affects the purchase decision of consumers. The marbling grade of beef is highly positively correlated with intramuscular fat content, but the traditional methods of increasing intramuscular fat, such as prolonging the fattening period and increasing the proportion of concentrates, are time consuming and costly. Studies showed that vitamin A can inhibit intramuscular fat synthesis in beef cattle, and the possible reasons attributed to the inhibiting action of vitamin A are given as follows: first, retinoic acid, a metabolite of VA, can regulate the differentiation of adipocytes. It has been reported that retinoic acid can inhibit the differentiation of a 3T3 cell line from preadipocytes to mature adipocytes; therefore, VA and its derivatives can inhibit the differentiation and maturation of intramuscular fat (21, 22). In addition, retinoic acid can regulate the expression of a growth hormone gene (23). Akazawa et al. (24) reported that the level of growth hormone in rats lacking VA was low. Akio et al. (25) measured the content of growth hormone in Japanese black and bovine serum. The results of their study showed that a low level of VA significantly affected the normal content of growth hormone, and some experiments showed that a low level of growth hormone could reduce the content of meat. As regards the marbling grade of cattle, retinoic acid may indirectly regulate the deposition of intramuscular fat by increasing the expression of growth hormone gene (25). However, the outcomes of similar investigations were heterogeneous (12–18). This study aimed to primarily investigate the effect of vitamin A on intramuscular fat in steers by conducting a meta-analysis.

In this meta-analysis, we first endeavored to complete a detailed and comprehensive literature search in the MEDLINE and Ovid databases to retrieve as many related studies as we could. Two independent reviewers screened the titles, abstracts, and full text of the articles and undertook the process of data extraction. In addition, heterogeneity in the studies included was assessed. Nevertheless, the results displayed significant heterogeneity (*p* < 0.01) in the studies enrolled. We planned to perform a subgroup analysis and meta-regression to explore the potential source of heterogeneity in the studies enrolled. Unfortunately, we were not able to complete these analyses due to the limited number of studies eligible for the inclusion criteria. Deek's funnel-plot asymmetry tests for publication bias revealed that there was no potential publication bias in the meta-analysis of IMF percentage and IMF score. Sensitivity analysis indicated that the pooled outcomes were robust after omitting one study after another in this meta-analysis. The present study showed that the addition of vitamin A reduced the percentage of intramuscular fat and increased intramuscular fat score in steers. Nevertheless, both the outcomes did not reach a statistical significance. The possible reasons for not achieving statistical significance may be attributed to the different numbers of studies included in each pooled analysis or other confounders, including the type of study design and steers' characteristics. This needs further investigation. Furthermore, significant statistical heterogeneity, which cannot be ignored in the interpretation of the present findings, was detected in this study.

Despite the limitations of the current study, we conclude that the addition of vitamin A could decrease intramuscular fat in cattle steers. More well-designed trials are needed in the future.

## Data availability statement

The original contributions presented in the study are included in the article/Supplementary material, further inquiries can be directed to the corresponding author.

## Author contributions

WL and FW conceived and designed this study. FS, YQ, CL, and YH were responsible for the collection, extraction, and analysis of the data. WL, FW, HW, BJ, and PZ were responsible for writing the article. JW, XS, MH, and DD polished the English language. All authors and participants reviewed the article and approved the final manuscript.

## Supplementary material

Supplementary Figure 1 Risk of bias graph.

Supplementary Table 1 Study characteristics. NR, not reported.

## References

[1] Pethick DW Harper GS Oddy VH. Growth, development and nutritional manipulation of marbling in cattle: a review. Aust J Exp Agric. (2004) 44:705–15. 10.1071/EA02165

[2] Shiranita K Hayashi K Otsubo A Miyajima T Takiyama R. Grading meat quality by image processing. Pattern Recognit. (1997) 63:3524–9. 10.1299/kikaic.63.3524

[3] Wood JD Enser M Fisher AV Nute GR Sheard PR Richardson RI . Fat deposition, fatty acid composition and meat quality: a review. Meat Sci. (2008) 78:343–58. 10.1016/j.meatsci.2007.07.019 22062452

[4] Peng DQ Smith SB Lee HG. Vitamin A regulates intramuscular adipose tissue and muscle development: promoting high-quality beef production. J Anim Sci Biotechnol. (2021) 12:34. 10.1186/s40104-021-00558-2 33663602 PMC7934359

[5] Chen W Chen G. The roles of vitamin a in the regulation of carbohydrate, lipid, and protein metabolism. J Clin Med. (2014) 3:453–79. 10.3390/jcm3020453 26237385 PMC4449691

[6] Palace VP Khaper N Qin Q Singal PK. Antioxidant potentials of vitamin A and carotenoids and their relevance to heart disease. Free Radic Biol Med. (1999) 26:746–61. 10.1016/S0891-5849(98)00266-4 10218665

[7] Tesoriere L Ciaccio M Bongiorno A Riccio A Pintaudi AM Livrea MA. Antioxidant activity of all-trans-retinol in homogeneous solution and in phosphatidylcholine liposomes. Arch Biochem Biophys. (1993) 307:217–23. 10.1006/abbi.1993.1581 8239660

[8] Oka A Maruo Y Miki T Yamasaki T Saito T. Influence of vitamin A on the quality of beef from the Tajima strain of Japanese Black cattle. Meat Sci. (1998) 48:159–67. 10.1016/S0309-1740(97)00086-7 22062888

[9] Gorocica-Buenfil MA Fluharty FL Bohn T Schwartz SJ Loerch SC. Effect of low vitamin A diets with high-moisture or dry corn on marbling and adipose tissue fatty acid composition of beef steers. J Anim Sci. (2007) 85:3355–66. 10.2527/jas.2007-0172 17709781

[10] Bryant TC Wagner JJ Tatum JD Galyean ML Anthony RV Engle TE. Effect of dietary supplemental vitamin A concentration on performance, carcass merit, serum metabolites, and lipogenic enzyme activity in yearling beef steers. J Anim Sci. (2010) 88:1463–78. 10.2527/jas.2009-2313 20023133

[11] Peng DQ Lee JS Kim WS Kim YS Bae MH Jo YH . Effect of vitamin A restriction on carcass traits and blood metabolites in Korean native steers. Anim Prod Sci. (2019) 59:2138–46. 10.1071/AN17733

[12] Harris CL Wang B Deavila JM Busboom JR Maquivar M Parish SM . Vitamin A administration at birth promotes calf growth and intramuscular fat development in Angus beef cattle. J Anim Sci Biotechnol. (2018) 9:55. 10.1186/s40104-018-0268-7 30062009 PMC6055337

[13] Knutson EE Menezes ACB Sun X Fontoura ABP Liu JH Bauer ML . Effect of feeding a low-vitamin A diet on carcass and production characteristics of steers with a high or low propensity for marbling. Animal. (2020) 14:2308–14. 10.1017/S1751731120001135 32517827

[14] Krone KG Ward AK Madder KM Hendrick S McKinnon JJ Buchanan FC. Interaction of vitamin A supplementation level with ADH1C genotype on intramuscular fat in beef steers. Animal. (2016) 10:403–9. 10.1017/S1751731115002153 26511067

[15] Kruk ZA Bottema MJ Reyes-Veliz L Forder REA Pitchford WS Bottema CDK. Vitamin A and marbling attributes: intramuscular fat hyperplasia effects in cattle. Meat Sci. (2018) 137:139–46. 10.1016/j.meatsci.2017.11.024 29182958

[16] Maciel FC Machado Neto OR Duarte MS Du M Lage JF Teixeira PD . Effect of vitamin A injection at birth on intramuscular fat development and meat quality in beef cattle. Meat Sci. (2022) 184:108676. 10.1016/j.meatsci.2021.108676 34656004

[17] Siebert BD Kruk ZA Davis J Pitchford WS Harper GS Bottema CD. Effect of low vitamin A status on fat deposition and fatty acid desaturation in beef cattle. Lipids. (2006) 41:365–70. 10.1007/s11745-006-5107-5 16808150

[18] Ward AK McKinnon JJ Hendrick S Buchanan FC. The impact of vitamin A restriction and ADH1C genotype on marbling in feedlot steers. J Anim Sci. (2012) 90:2476–83. 10.2527/jas.2011-4404 22307477

[19] Moher D Liberati A Tetzlaff J Altman DG. Preferred reporting items for systematic reviews and meta-analyses: the PRISMA statement. BMJ. (2009) 339:b2535. 10.1136/bmj.b2535 19622551 PMC2714657

[20] Cumpston M Li T Page MJ Chandler J Welch VA Higgins JP . Updated guidance for trusted systematic reviews: a new edition of the cochrane handbook for systematic reviews of interventions. Cochrane Database Syst Rev. (2019) 10:Ed000142. 10.1002/14651858.ED000142 31643080 PMC10284251

[21] Kawada T Aoki N Kamei Y Maeshige K Nishiu S Sugimoto E. Comparative investigation of vitamins and their analogues on terminal differentiation, from preadipocytes to adipocytes, of 3T3-L1 cells. Comp Biochem Physiol A Comp Physiol. (1990) 96:323–6. 10.1016/0300-9629(90)90699-S 1976474

[22] Kuri-Harcuch W. Differentiation of 3T3-F442A cells into adipocytes is inhibited by retinoic acid. Differentiation. (1982) 23:164–9. 10.1111/j.1432-0436.1982.tb01279.x 7166214

[23] Bedo G Santisteban P Aranda A. Retinoic acid regulates growth hormone gene expression. Nature. (1989) 339:231–4. 10.1038/339231a0 2716850

[24] Akazawa N Taniguchi K Mikami S. Effects of vitamin A deficiency on the function of pituitary-gonadal system in male rats. Nihon Juigaku Zasshi. (1989) 51:1209–17. 10.1292/jvms1939.51.1209 2513446

[25] Akio OKA Taiji DOHGO Masakatsu JUEN Takemitsu Gakkaiho SJNC. Effects of vitamin A on Beef Quality, Weight Gain, and Serum Concentrations of Thyroid Hormones, Insulin-like Growth Factor-I, and Insulin in Japanese Black Steers (1998).

