# Supplementary figures and images for: Effects of vitamin A on intramuscular fat development in beef cattle: A meta-analysis
Source: Front Vet Sci. 2023 Mar 15;10:1105754. doi: 10.3389/fvets.2023.1105754 (PMC10050684; doi:10.3389/fvets.2023.1105754)

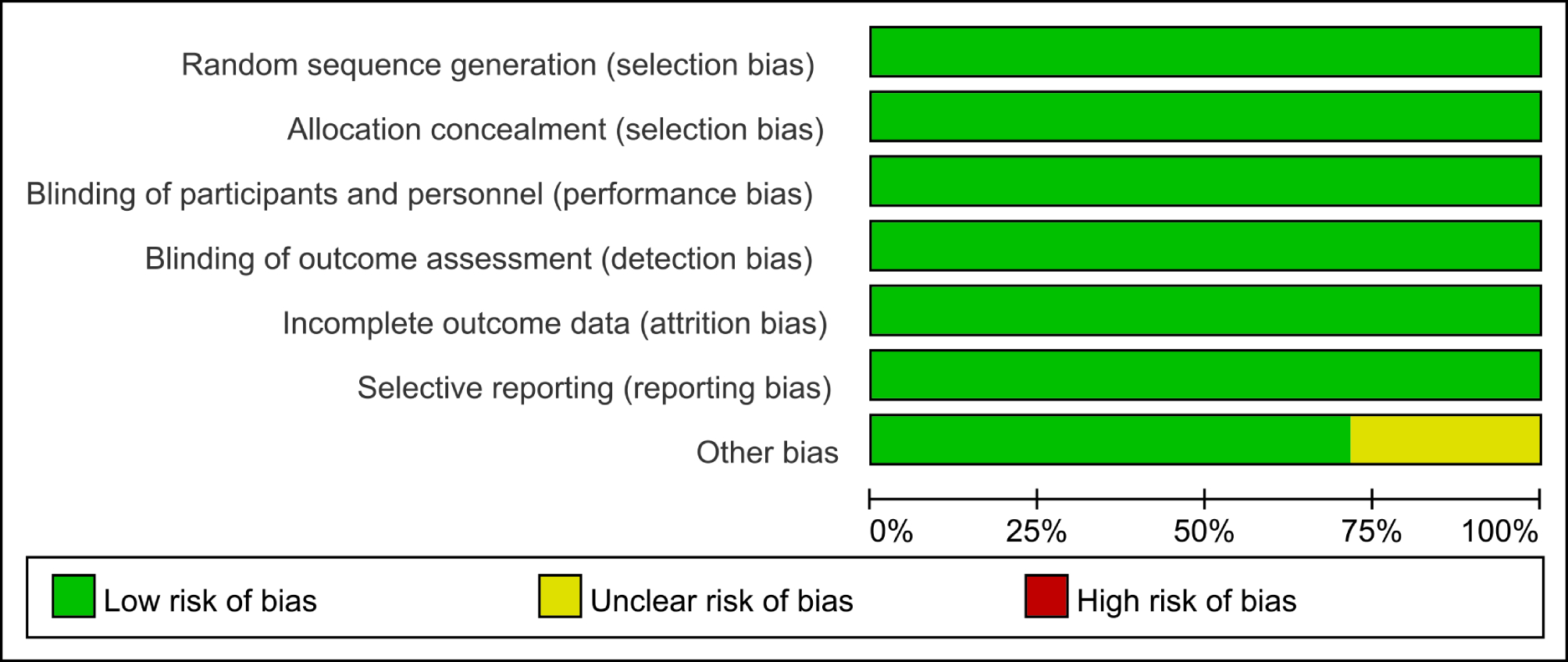

Supplement: Supplementary Figure 1 — Risk of bias graph. [file Image_1.JPEG]
