# Supplementary material for: Effects of vitamin A on intramuscular fat development in beef cattle: A meta-analysis
Source: Front Vet Sci. 2023 Mar 15;10:1105754. doi: 10.3389/fvets.2023.1105754 (PMC10050684; doi:10.3389/fvets.2023.1105754)
Supplement: Supplementary Table 1 — Study characteristics. NR, not reported. [file Table_1.DOCX]

**Table 1** Study characteristics

| **Name of first author** | **Year of publication** | **Type of steer** | **Study design** | **Total number of steers** | **Weight of steers at the start (mean±SD)** | **Addition of vitamin A** |
| --- | --- | --- | --- | --- | --- | --- |
|  |  |  |  |  |  |  |
|  |  |  |  |  |  |  |
| Siebert | 2006 | 12-mon-old Angus steers | steers were allocated to two treatment groups: vitamin A nonsupplemented (A−) and vitamin A supplemented (A+), | 20 | 354.9 ± 6.1 | 60,000 IU /100 kg LW/d |
| Ward | 2012 | Black Angus-cross steers | Vitamin A supplemented and unsupplemented | 130 | 306 | 750,000 IU/mo |
| Krone | 2016 | Black Angus crossbred steers | Three vitamin A treatments were targeted to meet 25% (zero supplementation), 50% and 75% of the animals’ daily requirement (2200 IU Vitamin A/kg DM) as outlined by NRC (1996). | 117 | 327 + 52 | 25% (zero supplementation), 50% and 75% of the animals’ daily requirement (2200 IU Vitamin A/kg DM) |
| Harris | 2018 | Black Angus steers | Treatment was three levels of injection: control, low-level and high-level | 25 | NR | Treatment was three levels of injection: control (0 IU), low-level (150,000 IU) and high-level (300,000 IU) |
| Kruk | 2018 | Angus steers | two treatment groups, vitamin A supplemented (A+) and vitamin A non-supplemented (A−) | 20 | NR | Equivalent of 60,000 IU retinyl palmitate/ 100 kg/day |
| Knutson | 2020 | Angus and Simmental steers | Half of the animals were assigned to a LVA finishing diet containing, and the remaining half were assigned to a control diet | 64 | 337.2 ± 5.9 | Half of the animals were assigned to a LVA finishing diet containing 723 IU vitamin A/kg of DM (Table 1), and the remaining half were assigned to a control diet (CON) consisting of the LVA diet supplemented with 2200 IU of vitamin A/kg of DM for a total of 2723 IU vitamin A/kg DM. |
| Maciel | 2022 | F1 Montana × Nellore calves | The animals were allotted into a completely randomized 2 × 2 factorial design (with or without vitamin A injection and males or females), consisting of the following treatments: ten male and seven female calves were treated with vitamin A, while 17 (10 males and 7 females) were not. | 34 | NR | The calves treated with vitamin A were given a dose of 300,000 IU of vitamin A |

NR, not reported.
